# The Nursing Organisational Context and the Surgical Site Infections: A Structural Equational Model

**DOI:** 10.1002/nop2.70372

**Published:** 2025-11-24

**Authors:** Eva Cappelli, Francesco Zaghini, Alessandro Sili, Jacopo Fiorini

**Affiliations:** ^1^ Department of Diagnostic and Public Health University of Verona Verona Italy; ^2^ Department of Biomedicine and Prevention University of Rome tor Vergata Rome Italy; ^3^ Nursing Department Tor Vergata University Hospital Rome Italy

## Abstract

**Aim:**

To assess the relationships existing among variables of the organisational context—staffing, stress‐related demands, workloads—and the occurrence of Surgical Site Infections (SSIs).

**Design:**

An observational study was conducted.

**Methods:**

A convenience sample of nurses directly involved in patient care was recruited. Staffing levels, workload and stress were measured using valid and reliable instruments. The prevalence of SSIs was recorded over 30 days using the European Centre for Disease Prevention and Control (ECDC) classification. Data were analysed using structural equation modelling.

**Results:**

This study enrolled 133 nurses. Higher SSI rates were associated with inadequate nurse staffing during daily shifts, increased workloads, evolving patient care needs and elevated stress levels among nurses due to work demands.

**Discussion:**

While the prevention of SSIs requires multidisciplinary collaboration, findings from this study suggest that the nursing organisational context—especially staffing and workload—had a direct impact on nurses' stress levels and an indirect influence on the prevalence of SSIs.

**Conclusion:**

Nurse managers and head nurses should improve nurses' working conditions and promote specific interventions for their organisational well‐being to influence SSI prevalence.

**Patient or Public Contribution:**

Despite patients and the public not being involved in the study's design and interpretation, the findings suggest new insights for healthcare organisations. Managing staffing levels, workloads and stress levels among nurses could improve patient safety and reduce the rates of SSIs. These outcomes advocate for strategic planning and organisational changes to boost the quality of care in surgical environments.

## 1 Introduction

Healthcare‐associated infections (HAIs) have been identified by the World Health Organisation (WHO) and the European Centre for Disease Prevention and Control (ECDC) as a global health challenge (Zingg et al. 2019). Surgical site infections (SSIs), the most frequent HAI, occur at or near the incision site within 30 or 90 days post‐implant surgery (Allegranzi et al. 2016; ECDC 2023). SSIs have substantial implications for patients and healthcare systems (ECDC 2023). When they are identified, further diagnostic tests and antibiotic treatment are required, often necessitating hospital readmissions and intensive care (ECDC 2017). SSIs also affect patients' lives, causing prolonged isolation from family members and work colleagues (Costabella et al. 2023).

**FIGURE 1:**
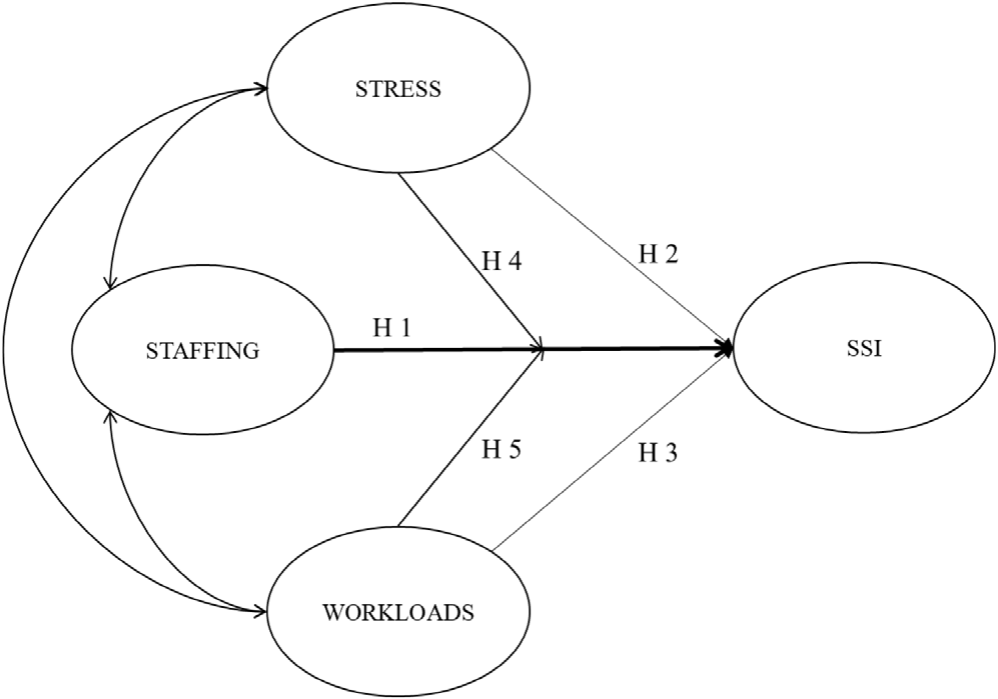
Model of research hypothesis. SSI, surgical site infection.

The WHO (2016) guidelines for SSI prevention lack the organisational strategies needed to manage SSI risk factors (Zingg et al. 2015). SSI prevention is a complex and multidimensional process, requiring adequate staff training and expertise to understand the risks associated with the type of surgery and the patient's clinical condition, and to implement effective SSI prevention and control (WHO 2016). The implementation of evidence‐based care practices, such as preoperative patient body wash (Jurt et al. 2021), perioperative glycaemic control, antimicrobial prophylaxis and institutional and formal nurses' training decreased SSI incidence by up to 60% (Horgan et al. 2023). It is not yet clear what influence staffing characteristics, work organisation and the well‐being of nurses can have on the SSI development (Tvedt et al. 2017). This study aimed to use a multidimensional model (Figure 1) to assess the relationships that exist among some organisational context variables such as staffing, stress‐related demands, workloads and SSIs.

## 2 Background

Research shows that HAIs are associated with clinical and working context variables. Clinical variables include the patient's condition, the type of surgery and the antibiotic treatment administered (Allegranzi et al. 2016; ECDC 2023). Working context variables include the level of nursing staffing, which evidence suggests should be 1:6 (nurse:patient) (Aiken et al. 2024), the nursing leadership style (Cappelli et al. 2024; Cummings et al. 2021), technological innovation (Mitchell et al. 2018) and the organisational context (van Buijtene and Foster 2019; Zingg et al. 2015). Nurses' education and skills are other determinants in HAI development; thus, a low rate of HAIs is observed when patients are cared for by a clinical nurse specialist, or a highly trained and experienced nurse (e.g., wound care nurse, or nurse practitioner) (Mitchell et al. 2018). In this regard, Zingg showed that high bed occupancy, inadequate staffing levels, unbalanced workload regarding resource availability and poor availability of equipment and materials affected the Infection Prevention Control Program (IPC) implementation (Zingg et al. 2015).

## 3 Methods

### 3.1 Aims and Hypotheses

Despite this evidence, the literature often provided aggregated HAI data, without differentiating by typology and investigating the impact of contextual variables on HAIs (Mitchell et al. 2018). For example, while literature focuses on the surgical nurse's knowledge and training to reduce SSI occurrence (Horgan et al. 2023), the relationship between nurses' shift staffing and SSI occurrence is unclear. Given the above, it could be hypothesised that (H1) there is a relationship between staffing levels (patient–nurse) and SSIs.

In recent years, nurses have managed an increasing number of additional organisational demands. These demands included professional responsibilities for dealing with COVID‐19, following institutional duties on documentation and adhering to institutional policies and protocols, which, combined, severely influenced nurses' lives (Zaghini et al. 2020). Initially, nurses could cope with the challenging working conditions, completing the required tasks and investing heavily in their psychophysical resources (Zaghini et al. 2020). However, over time, these coping strategies did not compensate for the organisational demands; thus, nurses' stress increased and job satisfaction and quality of care decreased (Wenderott et al. 2023; Zaghini et al. 2020). Therefore, it would be interesting to understand how nurses' psychophysical conditions, due to the organisational demands and working conditions, might affect SSI development. It could be hypothesised that (H2) there is a relationship between working demands, nurses' psychophysical condition and SSIs.

The surplus workload for nurses could depend on various factors, such as the patient's clinical condition, caring time, new technologies, the nurse–patient ratio, turnover (Ivziku et al. 2022) and the preventive measures against HAIs (Giuliani et al. 2018). Recognising and containing SSIs requires skilled and competent nurses (ECDC 2017), access to necessary equipment and tools and enough staff to effectively care for patients (Peutere et al. 2023). Organisations that fail to understand their working contexts and implement strategies to deal with the dynamic flow between the organisational demands and nurse shifts in each care setting, could lead us to hypothesise that (H3) there is a relationship between nurse workload and SSIs (Figure 1).

### 3.2 Study Design

A single‐centre observational study was conducted from September to November 2022. The checklist ‘Strengthening the Reporting of Observational Studies in Epidemiology’ (STROBE) was used to conduct and report this study (Vandenbroucke et al. 2007).

### 3.3 Study Setting

The study was conducted in a 1200‐bed Italian hospital, where nurses followed a shift schedule divided into time slots: morning (7:00 AM–2:00 PM), afternoon (2:00 PM–9:00 PM) and night (9:00 PM–7:00 AM). The hospital SSI protocol included prevention, identification, management, staff training and education, patient education, use of technology and audit conduction. The protocol was based on the ECDC recommendations for the management of SSIs (ECDC 2023) and was clinically implemented in January 2022 by the infection control nurses and head nurses. Ward nurses were responsible for SSI prevention, management, treatment and follow‐up.

### 3.4 Sampling

A sample of nurses working in different wards (oncology, surgery and medicine) was enrolled. Nurses who voluntarily consented to participate had to have worked in direct contact with patients for at least 6 months. Novice nurses in the organisation (working experience of less than 6 months), leaders and nurses' managers were excluded from the study.

### 3.5 Ethical Considerations

The study followed the Declaration of Helsinki principles (World Medical Association 2024) and was approved by an Ethics Committee (Prot. No. S00008/2022). All participants were assigned an alphanumeric code to ensure anonymity, and data were analysed in aggregate form. Online data storage was created and protected by a double identification system (personal ID and secure code) accessible only to the researchers, and a security system monitored unauthorised access, ensuring data protection and confidentiality.

### 3.6 Data Collection

A web survey, via the participant's hospital email, was used to collect responses. About 164 emails were sent with weekly reminders. The first page of the survey described the study aim and requested informed consent. The questionnaire was fully anonymous and completed once by each participant. Additionally, staff levels and SSI prevalence were collected over 1 month.

### 3.7 Instruments

Data were collected using two tools. The first, the Nurse Questionnaire, was a 2‐part web survey. The first section collected the participants' sociodemographic and work‐related data, and the second used two validated scales to evaluate perceived workload and work‐related stress.

The Quantitative Work Index (QWI) assessed perceived workloads through four items, with a Cronbach's alpha (*α*) of 0.82 in the previous study (Barbaranelli 2013; Spector and Jex 1998) and 0.89 in this study.

The Health Safety Executive Indicator tool (HSE‐IT) evaluated perceived work‐related stress through 19 items, with *α* of 0.85 in the previous study (Marcatto 2011) and 0.86 in this study.

The second tool (Outcomes Form) was a specially developed form to collect SSI information and nursing staffing levels. It was filled in daily by each ward head nurse for 30 consecutive days detailing the number of nurse staff on the three scheduled shifts and the SSI prevalence (Fiorini et al. 2022). SSIs were diagnosed based on specific symptoms described by the ECDC (2017). Head nurses were trained to correctly complete the form ahead of data collection.

### 3.8 Statistical Analysis

The sociodemographic data, working characteristics and instrument measurements were analysed using descriptive (mean and standard deviation SD) and inferential statistics (Pearson's correlation and linear regression). The instruments' internal consistency was estimated through Cronbach's *α* (Nunnaly 1994). To test the percentage of variance shown by the independent variables (staffing, workloads and stress) versus the dependent variable (Surgical Site Infections), a linear regression test with the step‐back method was used. To test the hypotheses under study, a Structural Equation Model (SEM) with reliability correction for the one‐dimensional variables was constructed using the Maximum Likelihood (ML) method and was conducted at ward level, pairing nurses' responses to SSI prevalence. The fit indices considered acceptable were chi‐square (not significant), RMSEA (< 0.06), CFI (> 0.90), TLI (> 0.90) and SRMR (< 0.08) (Muthén and Muthén 2017). SPSS Ver 22 and MPlus Ver 7.1 were used for analysing data, setting the significance level at *p* < 0.05.

## 4 Results

### 4.1 Sample and Organisational Characteristics

The sample, with a response rate of 81%, consisted of 133 nurses, mainly women (*N* = 105; 78.9%) with a mean age of 37 (SD = 9.6). Most of the nurses (*N* = 101; 75.9%) had a bachelor's degree in nursing and were married (*N* = 59; 44.4%). Sixty‐three per cent of nurses worked in the medical area (*N* = 84), 21% in the surgical area (*N* = 28), and 16% worked in the oncology ward (*N* = 21). Nurses worked a mean of 8.4 h daily (SD = 5.5) and had worked as a nurse for a mean of 11.68 years (SD = 8.8). Nurses perceived a workload level mean of 3.29 (SD = 1.11) and stress level of 2.78 (SD = 0.78).

Staffing levels (nurse/patient ratio) measured were found to be daily at a mean of one nurse for over seven patients (SD = 0.3). The staffing level for shift scheduling is described in Table 1. During the 30 days of data collection, a prevalence of 654 SSIs was measured. Specifically, 102 occurred in the neurology ward (15.60%), 43 in rehabilitation (6.57%), 153 in oncology (23.39%), 162 in surgery and hepatology (24.77%), 22 in nephrology (3.36%), 122 in the trauma centre (18.65%) and 50 (7.65%) in cardiology.

**TABLE 1:** Socio‐demographic and occupational characteristics of the sample (*N* = 133).

	*N*	%	*M*	SD
Age			37	9.6
Gender				
Male	28	21.1%		
Female	105	78.9%		
Non binary	—	—		
Marital status				
Single	68	51.1%		
Married	59	44.4%		
Separated/Divorced	6	4.5%		
Professional title				
University diploma	32	24.1%		
Bachelor's degree	101	75.9%		
Clinical setting				
Medicine	84	63%		
Surgery	28	21%		
Oncology	21	16%		
Working years			11.6	8.8
Daily working hours			8.4	5.5
Weekly extra working hours			13.6	34.7
Number of working absences for sickness in the last 6 months			3.9	7.2
Ratio staffing (nurses: patients)			1:7.1	0.3
Ratio staffing morning			1:5.3	0.3
Ratio staffing afternoon			1:7.7	0.3
Ratio staffing night			1:9.1	0.3

Abbreviations: *M*, mean; SD, standard deviation.

### 4.2 Correlations

A negative correlation was found between SSIs and staff levels (*r* = −0.51; *p* < 0.001). Stress (*r* = 0.26; *p* = 0.003) and workloads (*r* = 0.23; *p* = 0.007) were positively correlated with SSI (Table 2).

**TABLE 2:** Descriptive analysis, reliability and correlations among the studied variables.

Variable	*M*	SD	*α*	SSI	Staffing	Stress
SSI	11.88	12.03	—			
Staffing	0.14	0.03	—	−0.51**		
Stress	2.78	0.78	0.86	0.26*	−0.05	
Workloads	3.29	1.11	0.89	0.23*	−0.10	0.71**

Abbreviations: *α*, Cronbach's *α* coefficient; *M*, mean; SD, standard deviation; SSI, surgical site infection.

*Significance level *p* < 0.05; **Significance level *p* < 0.001.

### 4.3 Linear Regression

The regression model analysis was statistically significant (*R* ^2^ = 0.182; *p* < 0.001) and it showed that nurses' staffing (*β* = −0.338; *p* < 0.001), perceived workload and stress (*β* = 0.251; *p* = 0.002) influenced SSI prevalence.

### 4.4 Structural Equation Model (SEM)

**FIGURE 2:**
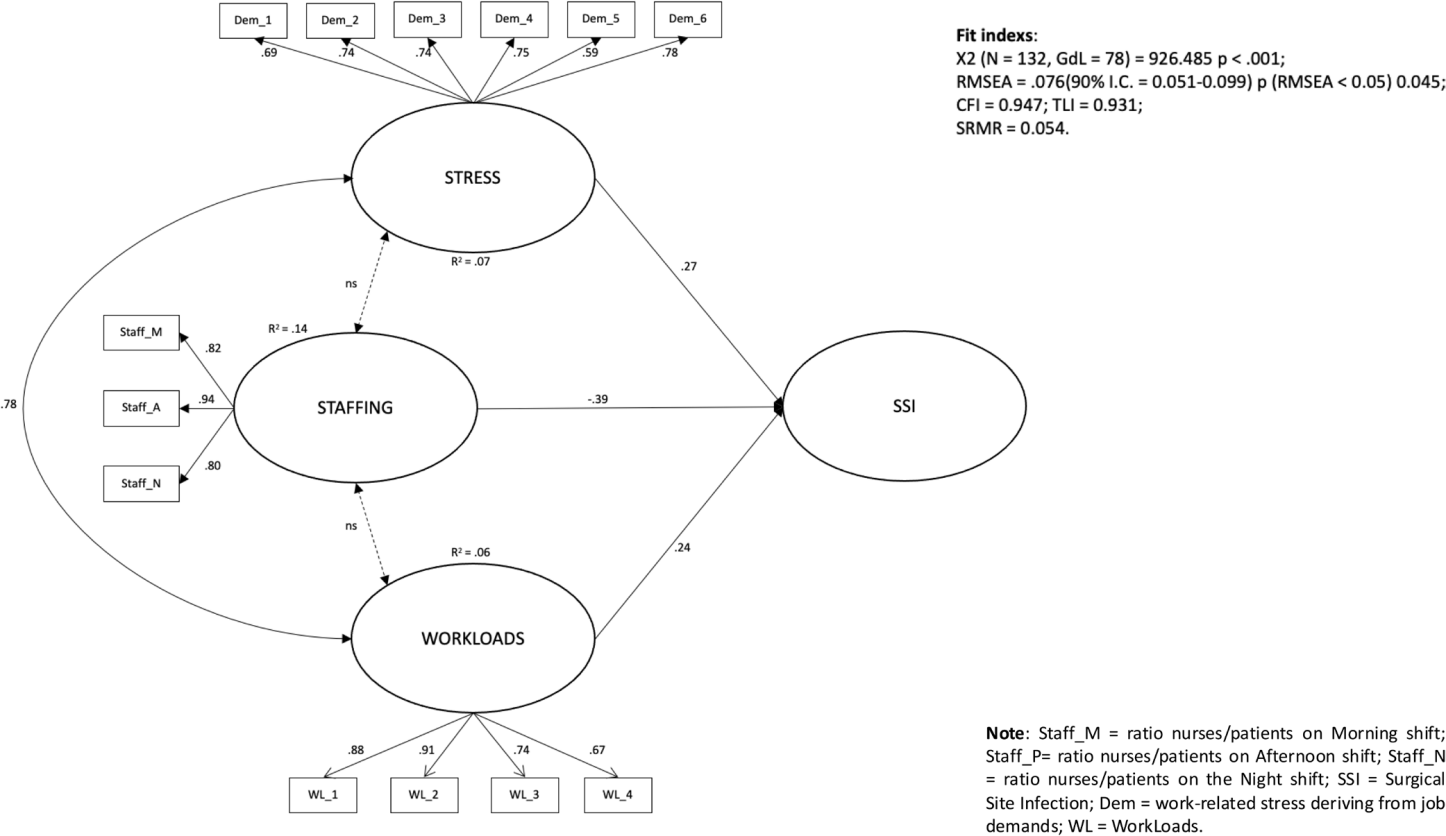
Structural equation model results. Dem = work‐related stress deriving from job demands; SSI = surgical site infection; Staff_M = ratio nurses/patients on morning shift; Staff_N = ratio nurses/patients on the night shift; Staff_P = ratio nurses/patients on afternoon shift; WL = workloads. Fit indexs: *χ* ^2^(*N* = 3132, df = 78) = 926.485 *p* < 0.001; RMSEA = 0.076 (90% CI = 0.051–0.099) p(RMSEA < 0.05) = 0.045; CFI = 0.947; TLI = 0.931 and SRMR = 0.054.

The SEM demonstrated a satisfactory model fit, as shown in Figure 2 (CFI = 0.947; RMSEA = 0.076; *χ* ^2^ = 926.485, df = 78, *p* < 0.001). Regarding the first research hypothesis, the nursing staff levels had a negative and direct association with SSIs (*β* = −0.39; *p* < 0.001). Moreover, nurses' stress deriving from the organisational demands was positively associated with SSIs (*β* = 0.27; *p* < 0.001), as hypothesised in the second hypothesis. Similarly, according to the third hypothesis, a positive and direct association was noted between nurse workloads and SSIs (*β* = 0.24; *p* < 0.001).

## 5 Discussion

This study aimed to assess the relationships existing among variables of the organisational context—staffing, stress‐related demands, workloads—and SSI occurrence. Results showed that an SSI increase can be determined by low staffing levels, high‐stress levels deriving from organisational demands and high workloads. A negative relationship between the nurse/patient ratio and HAI increase has already been studied (Mitchell et al. 2018). However, predictive values of the SEM model, focusing on SSIs, corroborated that the synergy between workloads, work demands and nurses' shift patterns contributed to SSI occurrence. In our study, only in the morning shifts (7 AM–2 PM) was the nursing staff well balanced in the nurse/patient ratio and adhered to the international ratio of 1:6 (Mitchell et al. 2018). Previous research demonstrated that a balanced nursing staff ensured more time for caring activities and specialist consultations, to review the patient's clinical condition (Congdon et al. 2020). In contrast, the staffing levels registered during the afternoon (2:00 PM–9:00 PM) and night (9:00 PM–7:00 AM) shifts were inadequate to the desired standard (Congdon et al. 2020). This work organisation could depend on the common belief that the caring activities were mostly performed in the morning rather than in other shifts (Congdon et al. 2020). During the afternoon and night, workloads were still significant and further aggravated by the activities unfinished in the morning, increasing workloads and stress due to these demands (Books et al. 2020). Guaranteeing the right staffing level should be a priority for the nursing manager to improve nurse job satisfaction (Wenderott et al. 2023) and quality of care (Dall'Ora et al. 2016).

The SEM results showed a positive association between workloads, stress deriving from organisational demands and SSI occurrence. As already found in previous studies (Chang et al. 2022; Zaghini et al. 2020) this study confirmed that high workloads and work‐related stress interfered with caring activities. With the increased workloads, nurses might dedicate less time to proper hand hygiene (Chang et al. 2022), surgical medication and drainage management (ECDC 2017; WHO 2016). This increased the risk of SSI (Books et al. 2020), due to missed nursing care and possible mistakes performed by nurses in clinical practice (Farnese et al. 2019).

Beyond the direct effects of workload on clinical performance, more evidence indicates that heavy workloads lead to nurse burnout by impairing alertness and reducing adherence to clinical protocols, ultimately affecting patient outcomes (Zaghini et al. 2020). Prolonged exposure to challenging organisational conditions results in emotional exhaustion, depersonalization, and a decreased sense of personal achievement, all core elements of burnout (Dall'Ora et al. 2016; Li et al. 2024), which impair compliance with evidence‐based practices (Zaghini et al. 2020). Burnout acts as a mediator between work environment and patient safety outcomes (Aiken et al. 2024; Dall'Ora et al. 2016), showing that supportive and transformational leadership (Cappelli et al. 2024), adequate staffing (Mitchell et al. 2018) and psychological safety reduce burnout (Zaghini et al. 2020) and improve adherence to IPC guidelines. Burnout thus represents a key indicator of organisational health with direct implications for clinical safety (Li et al. 2024). To address this issue, measures such as optimising nurse–patient relationships, reducing administrative burdens, clarifying roles and providing emotional support prove essential in protecting staff well‐being and strengthening infection prevention practices (ECDC 2017; Jun et al. 2021). In summary, prioritising organisational well‐being preserves nurses' mental health and operational efficiency, and promotes motivation and consistent adherence to infection prevention standards.

### 5.1 Implications of the Study

This study provides clear suggestions for both clinical and managerial practice. Despite efforts to keep SSI prevention strategies dynamic and responsive, the collaboration of the entire healthcare team is still necessary to effectively reduce SSI prevalence (ECDC 2017; Horgan et al. 2023). Therefore, it is vital that nursing managers take responsibility to organise and encourage their team to effectively manage SSIs, considering the implications of the organisational context and potential gaps in the complex patient care process (Farnese et al. 2019; Zaghini et al. 2020).

Specifically, the results provide insight on the motivations behind the team's well‐being (Cummings et al. 2021) and the principles (e.g., values and behaviours) of each healthcare worker (Farnese et al. 2019). The Magnet hospital's approach (Fischer and Nichols 2019) is a key example as it holds attention to safety and quality of services to the highest standard (Graystone 2019). Therefore, hospitals looking to reduce the prevalence of SSIs should take inspiration from the Magnet hospital, implementing dynamic healthcare models that consider the patient's clinical condition, and a flexible work organisation, acknowledging the skills and abilities of each healthcare worker (Tvedt et al. 2017).

Dedicated training for nurses promotes adherence to IPC practices, thus reducing SSI rates, also shown in the literature (Horgan et al. 2023). Training increases nurses' awareness of potential malpractice and their role in influencing the patient's clinical course of care.

This increased awareness permits nurses to influence and shape the organisational culture of the ward if supported by their head nurse and the entire organisation.

### 5.2 Limitations

The study results must be considered in light of some limits. A small number of nurses enrolled within a single hospital through convenience sampling might not allow for the generalizability of the results. Besides, this study did not consider nurses' skill mix, which might have influenced the results obtained. Workload and stress levels were not measured for each performed shift, only as general data. Furthermore, the self‐reporting instrument may have influenced the collection of participants' variables and SSI reporting. To address these limitations, future studies should enrol multiple hospitals using a large sample to improve generalizability. Collecting data on nurses' experience levels and professional qualifications could control skill‐related factors influencing SSIs. Using electronic diaries or wearable devices, real‐time or shift‐level monitoring of workload and stress could provide more accurate assessments of working conditions. Finally, combining self‐reported measures with objective data sources, such as staffing records, documented adherence to infection prevention protocols, and SSI surveillance data, would reduce response bias and strengthen the validity of the findings.

## 6 Conclusion

This research showed that adequate nurse staffing, reduced stress and manageable workloads improve adherence to IPC practices and reduce the prevalence of SSIs. The results emphasised the need for nurse managers to prioritise improving nurses' working conditions and well‐being, considering also available resources, demands of patient care and the organisation's limitations. Adequate staffing not only supports nurses' mental health but also plays a significant role in minimising preventable issues such as SSIs. Regarding the predictive nature of organisational factors on care outcomes, future studies should expand on these insights by including larger, more varied samples from different healthcare environments. Additionally, research should examine workload and stress on a shift‐by‐shift basis, include objective clinical outcomes and assess the impact of the nurses' different skills. These strategies could lead to more refined evidence to guide effective organisational interventions, ultimately improving staff well‐being, ensuring patient safety and reducing SSIs.

## Author Contributions

**Eva Cappelli:** methodology, investigation, visualisation, writing – original draft, data curation. **Francesco Zaghini:** conceptualization, methodology, visualisation, writing – original draft, data curation, investigation, formal analysis, software. **Alessandro Sili** and **Jacopo Fiorini:** conceptualization, methodology, visualisation, validation, data curation, writing – review and editing, supervision.

## Ethics Statement

The principles of the Declaration of Helsinki (World Medical Association 2024) have been followed and the Ethics Committee of the chosen hospital approved this study (Prot. No. S00008/2022). At the web survey's beginning, participants were informed about the study's purpose and requested informed consent. All data were collected, guaranteeing the complete participant's anonymity and analysing the data aggregately.

## Conflicts of Interest

The authors declare no conflicts of interest.

## Data Availability

The data that support the findings of this study are available on request from the corresponding author. The data are not publicly available due to privacy restrictions. There are two authors experts in statistics (F.Z. and J.F.) that have checked the data prior to submission.
